# Compact and reciprocal probe-signal-integrated rotational Doppler velocimetry with fiber-sculpted light

**DOI:** 10.1038/s41377-025-01747-8

**Published:** 2025-02-17

**Authors:** Zhenyu Wan, Ziyi Tang, Xi Zhang, Miles J. Padgett, Jian Wang

**Affiliations:** 1 Wuhan National Laboratory for Optoelectronics and School of Optical and Electronic Information, Huazhong University of Science and Technology, Wuhan, 430074 Hubei China; 2 Optics Valley Laboratory, Wuhan, 430074 Hubei China; 3 School of Physics and Astronomy, University of Glasgow, Glasgow, G12 8QQ UK

**Keywords:** Optical sensors, Imaging and sensing

## Abstract

In recent years, with the clarification of the mechanism of the rotational Doppler effect (RDE), there has attracted extensive attention to its development of applications, especially in the detection of the angular velocity of rotating objects. On the other hand, optical fiber technology is widely applied in laser velocimetry from beam delivery to scattered light collection, aiding the miniaturization of instruments. Here we report the first all-fiber rotational Doppler velocimetry (AF-RDV) with a single probe based on a fabricated mode-sculpted fiber-optic element. The constructed AF-RDV can be operated in two reciprocal schemes wherein exchanging the illuminating mode and detected mode. Using this, we experimentally demonstrate the mode-changing dependent nature of the RDE. Particularly, the results suggest that the rotational Doppler shift can be observed by mode-filtering the scattered signal even with a non-twisted probe light. We also show the achromatic property of the RDE by scanning the incident wavelength, enabling the AF-RDV within an ultra-broadband operation range. The AF-RDV exhibits favorable performance for detecting spinning rough surfaces. It may provide an exciting new practical sensing instrument with significant prospects for monitoring angular motion in both research and industry.

## Introduction

Optical vortices, the intrinsic properties of helically phased light beams, feature phase singularities that appear on a perpendicular plane of the beam axis, resulting in the phase indeterminate^1^. In general, optical vortices occur in a light beam with a screw phase dislocation of the form exp(*iℓθ*), where *θ* is the azimuthal angle of the cylindrical coordinates and *ℓ* is known as the topological charge^2^. Since it was recognized in 1992 by Allen et al. that a light beam with such spiral phase term carries a definite amount of orbital angular momentum (OAM) of *ℓ*ℏ per photon^3^, where ℏ is the reduced Plank constant, the extensive and in-depth research in this field has promoted its rapid development^4–6^. The OAM beams are characterized by helical wavefronts and appear as doughnut-shaped rings with a central intensity null. Another distinct feature of OAM beams is that the Poynting vector of the light field is twisted in a tiny inclination with the propagation axis, i.e., *ℓ*/*kr*, where *k* is the wave number and *r* is the off-axis radius^7^. Particularly, the optical vortices and OAM beams often refer to the spatial modes in the more general higher-dimensional structured light and multi-degrees-of-freedom light^8–10^. Light beams carrying OAM have attracted widespread attention during the last three decades in optical manipulations^11–13^, optical communications^14–18^, imaging^19–21^, quantum information^22–24^, and other fields^25–32^. For instance, the helical phase structure of OAM beams has enabled important applications in optical metrology and sensing^33^. Recently, OAM-based metrology and sensing have been widely investigated in various application scenarios, such as detecting shape parameters (e.g., contour or defect)^34–37^, property parameters (e.g., refraction index or chirality)^38–40^, motion parameters (e.g., rectilinear or angular velocity)^41–44^, and weak measurement^45–47^. In particular, the OAM spectrum is utilized as a powerful tool there to obtain the parameters of the target.

As well known, the Doppler effect, a universal wave phenomenon, is presented to explain the shift in the frequency of a wave caused by relative motion between the source and the observer. With the study of OAM, a new type of Doppler effect is proposed to describe the relationship between rotational motion and frequency shift of a wave, i.e., rotational Doppler effect (RDE)^48^. On one hand, the RDE shares a common origin with the conventionally linear counterpart^49^, and thus its physical mechanism can be revealed from the perspective of the photon and wave nature of light respectively, namely energy transfer or time-varying phase^50,51^; on the other hand, considering the crucial role of OAM in the RDE, the OAM spectrum method is also encountered in the analysis for this process of OAM-matter interaction^52^. In recent years, the RDE has been widely studied with tremendous progress in both optical vortices and acoustic vortices^53–63^. Furthermore, lots of applications to measure angular velocities of rotating objects based on RDE were reported after the work by Lavery et al. in 2013^64^. Subsequently, the rotational Doppler velocimetry (RDV) is developed on diverse occasions including surface, microparticle, and fluid^65–75^. The RDV exhibits great potential as an efficient technique in metrology.

Nevertheless, the currently established RDVs are commonly composed by discrete optical elements in a free-space configuration, which has some nuisances such as high investment, bulky size, and poor flexibility, resulting in huge challenges in real engineering applications and commercial products. As for the conventional laser Doppler velocimetry (LDV), to improve the integration of the system, the fiber-based elements are employed to supersede the discrete free-space devices by far, so as to develop an all-fiber LDV^76^. Inspired by this, the optical fiber architecture may provide a reliable alternative for RDV to improve its robustness in practical scenarios. The optical fiber has become an essential channel for the communication infrastructure since its invention. Especially, the optical fiber is considered as a sterling transmission medium supporting the vortex modes^77–79^, which has been widely applied in mode-division multiplexing^80,81^. In our previous work, it has been demonstrated that the fiber eigenmode is applicable to motion information acquiring of a rotating object^82^. However, such a measurement system is still not compact enough because complex devices are required to manipulate the light field. Fortunately, there are abundant fiber-based elements designed to sculpt the optical modes effectively^83^. This means that the RDV can be constructed by an all-fiber configuration, and we call this all-fiber RDV (AF-RDV), which may be developed into a novel instrumentation with promising applications.

In this work, we propose and construct a compact and flexible AF-RDV to measure the angular velocity of the spinning surface. The core element in the AF-RDV, an ultra-broadband mode-selective coupler (MSC), is fabricated with low insertion loss, acting as a bifurcated mode gate to implement mode sculpting and filtering. Different from the previously demonstrated RDV by just using a single vortex mode or superposed vortices, a Gaussian mode (non-OAM or zero-order OAM) and superposed OAM mode are alternated here to illuminate the surface, showing the similarity of the eventual effect so that the RDE is recognized to have reciprocal property. By means of that, the rotational Doppler shift exhibits independence of the OAM in the probe light, and in contrast, its determinant lies in the variation of OAM during the interaction between the probe light and spinning surface. Another distinct feature of the configuration is the single optical front head, which integrates the probe port and signal collection port to further reduce the complexity and cost of system. Significantly, the achromatic property of the AF-RDV is experimentally demonstrated through wavelength scanning over a wide spectrum range. The AF-RDV, which has a competitive advantage in commercial applications for its cost-effectiveness and compactness, may promote the industrial prospects of the RDE theory in sensing and metrology.

## Results

### Concept and principle

The RDE describes the phenomenon that a vortex beam encounters a frequency shift from the spinning or rotating motion of an object or the field itself. Here we consider a general case of probe light to illuminate the rotating rough surface. In cylindrical coordinates, the complex amplitude of the probe light can be expanded as a Fourier series with the orthogonal basis of helical harmonics^84,85^, i.e., ${E}_{p}(r,\phi )={\sum }_{n}{E}_{n}(r)\exp (in\phi )$, where ${E}_{n}(r)$ denotes the complex amplitude of the harmonic with *n*-order OAM mode, *r* is the radial coordinate, and *ϕ* is the angular coordinate. A simplification is taken for characterizing the rough surface which is assumed as a phase-modulated element with homogeneous reflectivity. This means that the scattered light by the rough surface would get an adjusted phase distribution $\Phi (r,\phi )$, compared to the probe light. Particularly, this phase modification is angular-dependent, and thus when the rough surface spins at an angular velocity Ω around its central axis, the azimuthal angle is modified at time, i.e., $\tilde{\phi }=\phi -\Omega t$. Based on the mode expansion method^52^, we analyze the RDE induced by the scattering interaction from the rotating rough surface. Ignoring the radial difference, the phase modulation of the rotating rough surface can be decomposed into Fourier expansion form along the angular direction, which is similar to the superposition of a series of spiral phase plates. We can thus express the modulation function of the rotating rough surface as $M(r,\tilde{\phi })=\exp [i\varPhi (r,\phi -\Omega t)]={\sum }_{m}{A}_{m}(r)\exp (im\phi )\exp (-im\Omega t)$, where ${A}_{m}(r)$ is the complex coefficient of the *m*-order spiral component with normalization ${{\sum }_{m}|{A}_{m}(r)|}^{2}=1$, and its modulus and argument parts stand for the weight and initial azimuth, respectively. The distribution of ${A}_{m}(r)$ is related to the geometric properties of the scatter, such as its roughness and embossed periodicity with rotationally symmetric notches. The scattered light can then be written by:

1 $${E}_{S}={E}_{p}(r,\phi )\cdot M(r,\tilde{\phi })={\sum }_{n}{\sum }_{m}{E}_{n}(r){A}_{m}(r)\exp [i(m+n)\phi ]\exp (-im\Omega t)$$

**Fig. 1:**
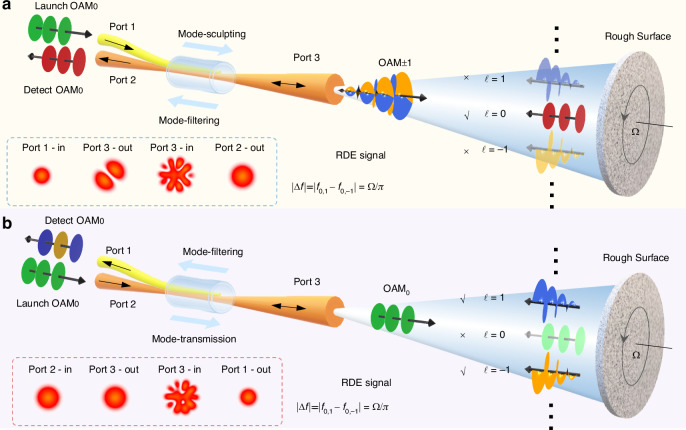
Concept and principle of all-fiber rotational Doppler velocimetry (AF-RDV). **a** Normal scheme of AF-RDV: the superposed vortices sculpted by fibers serves as the probe light, and the higher-order modes in the scattered light are filtered out in fibers for detecting the Gaussian components with rotational Doppler beat frequency. *f*_0,1_ and *f*_0,−1_ refer to the rotational Doppler shifts with OAM changing from ±1 to 0 order, respectively. **b** Reciprocal scheme of AF-RDV: the Gaussian mode serves as the probe light, and ±1-order OAM modes with rotational Doppler shifts are selected and reverse-sculpted in fibers into Gaussian modes to be detected. *f*_−1,0_ and *f*_1,0_ refer to the rotational Doppler shifts with OAM changing from 0 to ±1 order, respectively

One can see that the scattered light contains various vortex modes and each projected mode has a corresponding rotating Doppler shift depending upon the angular velocity Ω and the modulated mode order *m*. Generally, to realize the extraction of Ω, a key process in the RDV is mode sculpting and filtering. For example, a spatial light modulator (SLM) is commonly employed to reach this in previous configurations^64^. As a more compact solution, it is performed by a fiber element here in this proposed AF-RDV, i.e., the designed MSC, as shown in Fig. 1. The MSC is constituted by a single-mode fiber (SMF) and a multi-mode fiber (MMF) with three occupied ports, where port 1 and port 2 can be interchanged for launching the source and detecting the signal, respectively, while port 3 is probe-signal-integrated and simultaneously illuminates the rough surface and receives the scattered light. As a result, there would be two operating ways with different probe light, i.e., superposed vortices (OAM_±1_) and Gaussian mode (OAM_0_), as follows.

The first scheme is based on the superposed vortices interacting with a rotating scatter, like normal RDV in principle, as shown in Fig. 1a. When the Gaussian mode launched by port 1 is coupled to port 3, the output mode is sculpted to carry the ±1-order OAM. This corresponds to the *n* in Eq. (1) taking two values of ±1. The optical speckle scattered by the rough surface contains multiple modes. After being collected by port 3, the scattered signal will undergo mode filtering on its way to port 2. During this process, OAM_±1_ modes will be re-coupled back to port 1, while loss will mainly occur in the higher-order modes, so that ideally the detected signal in port 2 will carry a Gaussian mode primarily. The inset of Fig. 1a shows the simulated profiles of input and/or output beams at each port. The Gaussian mode selected from the scattered light means that $m+n=0$ in Eq. (1). Thus, the signal light at port 2 will consist mainly of two harmonics with the mode order change of −1 and +1 respectively, expressed as:

2 $$\begin{array}{l}{E}_{Sig(OA{M}_{\pm 1}\to OA{M}_{0})}={E}_{1}(r){A}_{-1}(r)\cdot \exp (i\Omega t)\\\qquad\qquad\qquad\qquad\quad+\,{E}_{-1}(r){A}_{1}(r)\cdot \exp (-i\Omega t)\end{array}$$

There is a frequency difference between the two harmonics in the complex amplitude of signal light, enabling a beat frequency that allows the photodetector (PD) to respond directly. The detected time-varying intensity signal can be written by:

3 $$\begin{array}{l}I{(t)}_{Sig(OA{M}_{\pm 1}\to OA{M}_{0})}={I}_{1,-1}+{I}_{-1,1}\\+\,2\sqrt{{I}_{1,-1}{I}_{-1,1}}\cdot \,\cos (2\Omega t+{\varphi }_{0})\end{array}$$

where ${I}_{1,-1}={|{E}_{1}(r){A}_{-1}(r)|}^{2}$ and ${I}_{1,-1}={|{E}_{-1}(r){A}_{1}(r)|}^{2}$ are the intensity of the two modulated harmonics, ${\varphi }_{0}={{\rm{arg}}}[{E}_{1}(r){A}_{-1}(r){E}_{-1}^{\ast }(r){A}_{1}^{\ast }(r)]$ is their initial phase difference, and the radial position is approximated to a constant value. As a result, the beat frequency of the detected signal light can be extracted by means of Fourier analysis as:

4 $${|\Delta f|}_{OA{M}_{\pm 1}\to OA{M}_{0}}=\Omega /\pi$$

This means that the beat frequency is positively correlated with the angular velocity when the probe light is sculpted with superposed OAM and the Gaussian mode is filtered from scattered light for detection.

Alternatively, another consideration is to use an unusual probe light with Gaussian mode, as shown in Fig. 1b. The launched Gaussian mode is transmitted directly from port 2 to port 3 via the MMF. As the probe light carries non-OAM, the *n* in Eq. (1) takes single value of 0. Then the modes in the scattered light are selected to be detected by the MSC with the simultaneous process of mode filtering and sculpting. Among the scattered modes, the Gaussian and higher-order components will return back to port 2 along the MMF and the efficient mode coupling from MMF to SMF in the designed MSC occurs within the 1-order mode group. As a result, the Gaussian and higher-order modes are filtered out, while the ±1-order OAM modes with Doppler shifts are reverse-sculpted into Gaussian modes to be detected. This means that $m+n$ may take two values of ±1 in Eq. (1). The resultant signal light at port 1 is expressed as:

5 $$\begin{array}{l}{E}_{Sig(OA{M}_{0}\to OA{M}_{\pm 1})}={E}_{0}(r){A}_{1}(r)\cdot \exp (i\phi )\exp (-i\Omega t)\\\qquad\qquad\qquad\qquad\quad+\,{E}_{0}(r){A}_{-1}(r)\cdot \exp (-i\phi )\exp (i\Omega t)\end{array}$$

One can see that this signal light also contains two harmonics with different rotational Doppler shifts, leading to an intensity beat signal as:

6 $$\begin{array}{l}I{(t)}_{Sig(OA{M}_{0}\to OA{M}_{\pm 1})}={I}_{0,1}+{I}_{0,-1}\\+\,2\sqrt{{I}_{0,1}{I}_{0,-1}}\cdot \,\cos (2\Omega t+{\varphi }_{1})\end{array}$$

where ${I}_{0,1}={|{E}_{0}(r){A}_{1}(r)|}^{2}$, ${I}_{0,-1}={|{E}_{0}(r){A}_{-1}(r)|}^{2}$, and ${\varphi }_{1}={\rm{arg}}[{A}_{1}(r){A}_{-1}^{\ast }(r)]$. The beat frequency in this detecting scheme is then determined by:

7 $${|\Delta f|}_{OA{M}_{0}\to OA{M}_{\pm 1}}=\Omega /\pi$$

Equation (7) indicates that the probe light without OAM can also induce rotational Doppler shifts once only specific non-zero OAM components are detected in the scattered light. This is similar in principle to a recent demonstration by Zhai et al.^62^. Comparing Eqs. (4) and (7), it is clear that the same relation reveals between the extracted Doppler beat frequency and the angular velocity in both cases. In other words, this shows the reciprocal property of the RDE. This means that the exchange of the modes in probe light and signal light would lead to the same rotational Doppler shift, and this further supports the fact that the RDE is dependent upon the mode changing rather than the initial or detected modes. Moreover, from Eqs. (4) and (7), we note that the rotational Doppler shift is independent of the wavelength of the probe light, which has been demonstrated in Lavery’s experiments before^86^. Hence, the AF-RDV is provided with an achromatic feature by using a wavelength-insensitive MSC with an ultra-broadband operating range.

### Implementation

**Fig. 2:**
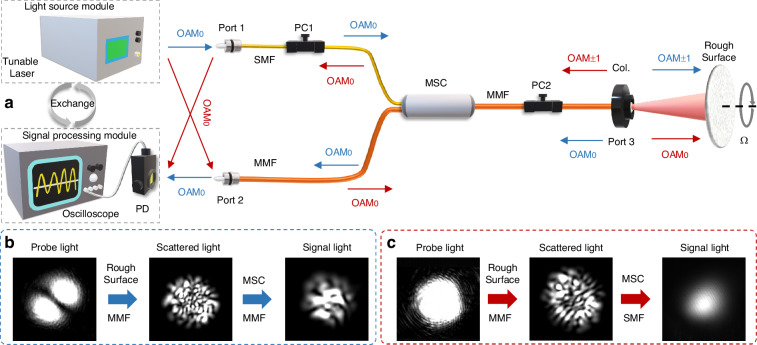
Experimental setup to demonstrate the AF-RDV. **a** Experimental setup with two operating schemes. The one is normal scheme that the light source module is connected to port 1 and signal processing module to port 2 (path along with blue arrows). Another is reciprocal scheme that the two modules are exchanged to connect the two ports (path along with red arrows). PC polarization controller, MSC mode-selective coupler, SMF single-mode fiber, MMF multi-mode fiber, Col. collimator, PD photodetector. **b**, **c** The measured intensity profiles of typical beams at 1550 nm for each of the two operating schemes

In the configuration of AF-RDV, the core element is the designed MSC, which is fabricated by splicing a standard SMF and an MMF with effective refractive index matching of desired modes by tapering process (see “Methods” for details). The experimental setup of AF-RDV is constructed by connecting each port of the MSC to each module, as shown in Fig. 2a. The light source module employed is a 10-mW tunable laser covering S, C, and L bands, and the signal processing module includes a PD and an oscilloscope to capture data and perform Fourier analysis. The two modules are respectively wired up to port 1 and port 2 of the MSC, and in particular, corresponding to the two operating schemes in the concept, the two modules can be exchanged with each other to launch different probe lights in port 3. Two polarization controllers (PC) are used to properly adjust the polarization in two fibers to ensure that the MSC is at the expected operating polarization. The AF-RDV has only a single probe composed of a collimator (Col.) with inserted port 3, which can combine the function of probe illuminating and signal collecting, enabling the greatly improved compactness of the system. The end of the MMF at port 3 is cut slantly to reduce the end reflection, thereby preventing unwanted noise caused by homodyne interference. The collimator chosen here has a long focal length to output a larger size of probe beam. The probe light is collimated and expanded to illuminate the spinning rough surface with normal incidence and center alignment.

When the probe light is considered to adopt the superposed vortices, the laser source is connected to port 1 and the PD to port 2. The OAM_0_ mode is then coupled from the SMF to MMF, turning into the OAM_±1_ mode with a petal-shaped profile. The signal scattered by the spinning rough surface is collected into the MMF by the collimator, and after mode filtering by the MSC, the OAM_0_ mode is mainly detected at port 2. The beams taken at 1550 nm are shown in Fig. 2b. It can be seen that the signal light exhibits intensity fringes, which are caused by the interference of the Gaussian mode and some remaining lower-order modes (for example, mainly the 2-order), where the Gaussian mode occupies most of the power and the higher-order components in the scattered speckle-like signal are notably filtered out through the tapered region of MSC. The fringes also suggest that OAM_±1_ modes are rarely there in detected signal light. In the reciprocal operation, when the probe light is just a Gaussian mode, the Gaussian beam launched by the laser source is switched in port 2 and the PD receives the signal light from port 1. After the scattered light is collected into port 3, the ±1-order OAM modes are selected for reverse-sculpting back to the Gaussian mode and coupled into the SMF for detection. As shown in the beams in Fig. 2c, the signal light appears as a Gaussian mode with high purity due to the constraints of boundary conditions in the SMF. In a sense, the MSC is regarded as a mode bifurcated gate in the system to perform mode sculpting, filtering and transmission functions. It allows the correct mode to be detected on one path and the unwanted mode to be dissipated on the other. This helps to suppress the rotational Doppler shifts carried by other idle modes in the scattered signal. As a result, there would be a narrow peak in the frequency spectrum of the signal light that stands out from the other peaks and the noise, and the angular velocity of the spinning rough surface can be deduced from the peak frequency.

### Experimental demonstration on the reciprocal property of RDE

**Fig. 3:**
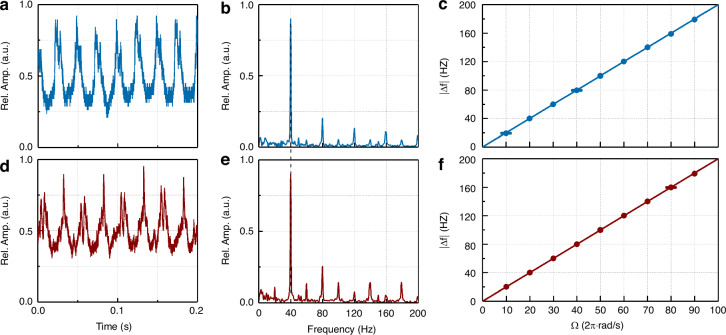
Experimental demonstration on the reciprocal property of rotational Doppler effect (RDE). **a**–**c** The probe light carries superposed vortices (OAM_±1_) and the Gaussian mode (OAM_0_) in scattered light is selected to be detected. **d**–**f** The probe light carries Gaussian mode (OAM_0_) and the OAM_±1_ modes in scattered light are selected to be detected. **a**, **d** The collected intensity signals and **b**, **e** the calculated Fourier frequency spectra under the rough surface spinning at 40π rad/s. **c**, **f** The measured beat frequency with an identical linear relation of the angular velocity in both schemes. The laser is tuned at a wavelength of 1550 nm

We first demonstrate the reciprocal property of RDE by exchanging the illuminating mode and the detected mode. The spinning rough surface measured here is a piece of slightly embossed tinfoil attached to a motor. In the experiment, the laser is tuned at a wavelength of 1550 nm. The received optical power by PD placed at port 2 gets a loss of about 27 dB compared to the light launched from port 1, and when the two are exchanged, the loss is about 30 dB. The switchable amplified PD (Thorlabs, PDA50B2) is set the gain step to 30 dB. The typical results are obtained by setting the rotational speed of the motor to 20 revolutions per second (rps), as shown in Fig. 3a, b, d, e, where the intensity signals are partly displayed with a total signal acquisition window of 1 s in the time domain, giving the frequency resolution of 1 Hz in fast Fourier transform (FFT). The relative amplitude in the figures is given by normalization with 0.9 to the maximum amplitude. In both schemes, the intensity signals show the similar periodicity, and their Fourier frequency spectra present the same maximum peak at 40 Hz. From Eqs. (4) and (7), it can be deduced that the angular velocity Ω is measured to be 40π rad/s, which is exactly consistent with the rotational speed set by the motor.

The rotational speed of the motor is then gradually adjusted from 10 to 90 rps with a 10 rps interval. It can be seen that the beat frequencies measured in both schemes get an identical linear trend of growth, as shown in Fig. 3c, f. This means that the RDE is reciprocal, i.e., the mode exchange between probe light and detected signal would lead to the same Doppler shift, even if there is a Gaussian beam without OAM to illuminate the object. This linear relationship between the beat frequency and angular velocity is in line with the theoretical predictions, enabling the detection of rotating scatter. The sensitivity of the AF-RDV is related to the topological charge of OAM in the mode sculpting/filtering, where for the mode carrying ±1-order OAM, the sensitivity for angular velocity sensing is 1/*π* Hz/(rad/s). The relative uncertainty of measurement accuracy is estimated to be 0.35% and 0.20% respectively for the two cases in Fig. 3c, f.

Note that in Fig. 3b, e, there are a few idle equidistant harmonic peaks in the Fourier spectra beyond the dominant Doppler peak. Particularly, the harmonic peaks correspond to different mode changes between illumination and detection. They originate from two factors, including slightly misaligned illumination (i.e., factor *n* in Eq. (1)) and other scattered modes in the initial speckle signal (i.e., factor *m* in Eq. (1)). On one hand, the spectrum of OAM components coaxial with the rotation axis of the scatter is broadened when the probe light is misaligned with respect to the rotation axis^87^. This suggests that the factor *n* in Eq. (1) would get some different values that deviate from the initial on-axis setting, that is, the misaligned probe beam is composed of some discrete modes with different *n*. On the other hand, the geometric characters of the rough surface would also lead to the decomposed spiral components of its modulation function, i.e., the discrete distribution of factor *m* in Eq. (1). For the AF-RDV, according to Eq. (1), the value of *m* + *n* denotes the mode order that the scattered light can take, and the amplified order of the rotational Doppler shift is determined by the *m*. As the *n* and *m* are expanded into discrete distributions, it would result in multiple harmonic components of rotational Doppler shift in the scattered mode. Indeed, these regularly discrete peaks do not imply a reduction in the signal-to-noise ratio (SNR) of the system because they are actually the idle signals rather than noise, while the source of noise here is mainly shot noise and thermal noise during weak signal detection.

### Achromatic results with wavelength scanning

**Fig. 4:**
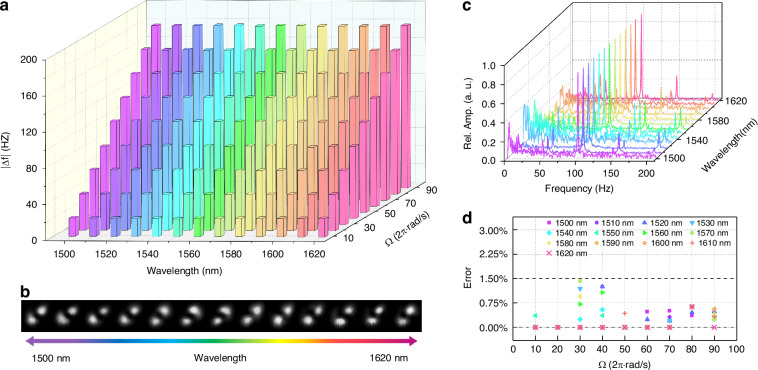
Measured results for achromatic performance of the AF-RDV under wavelength scanning. **a** The measured Doppler beat frequency versus different angular velocities and operating wavelengths. **b** The intensity profiles of the probe light carrying OAM_±1_ mode sculpted by the MSC under light launching at wavelengths from 1500 to 1620 nm with a 10 nm interval. **c** The Fourier frequency spectra of the signal light at different wavelengths under the rotational speed Ω = 100π rad/s. **d** The error estimation of (**a**)

The achromatic performance of the AF-RDV across an ultra-broadband wavelength range is experimentally demonstrated by wavelength scanning of the tunable laser, as shown in Fig. 4. In the experiment, the tunable laser is modulated from 1500 and 1620 nm with a 10 nm interval. The laser is connected to port 1 in Fig. 2a so the probe light here is superposed vortices. The intensity profiles of the superposed vortices sculpted by the MSC under excitation of different wavelengths are presented in Fig. 4b. The invariant beams indicate that such device has a favorable broadband performance. Typically, we set the rotational speed of the rough surface to 50 rps and obtain the Fourier frequency spectra of the signal light at different wavelengths, as shown in Fig. 4c. It is clear that all the spectra get their maximum peak at 100 Hz. The relation between the measured peak frequency and the set angular velocity satisfies Eq. (4). This demonstrates the wavelength-insensitive property of the RDE from another perspective, similar to the observation with white-light illumination^86^. Furthermore, we measure the Doppler beat frequency by varying the rotational speed and tuning the wavelength respectively, as shown in Fig. 4a. One can see that, at each wavelength the measured beat frequency increases with the same linear trend as the increase of rotational speed (each column), and at each rotational speed the measured beat frequency remains the same value for different wavelengths (each row). Each data bar in Fig. 4a is given by averaging 7 measurements, and the corresponding error deviating from the theoretical expectation is shown in Fig. 4d. The maximum error margin of the AF-RDV within its operating band is estimated to be less than 1.5%, and there is no evident correlation between the error value and the operating wavelength. This means that the constructed AF-RDV has excellent achromatic performance. In other words, the measurement accuracy of the system would not get impact by factors such as the laser linewidth and temperature drift, and thus, the cost of the system setup and maintenance could get greatly reduced.

## Discussion

Generally, there are two ways to implement the RDV, for instance, by using superposed two OAM modes with different topological charges or by setting up an interferometric system with an extra reference beam. In the AF-RDV, we adopt a fringe-detected configuration, where a probe light is simply required and the two vortex modes with opposite helicity are coaxially transmitted. Therefore, compared to the homodyne configuration, such technique is more resistant to the environmental disturbance. Besides, thanks to the fiber-based elements, the AF-RDV can be constructed highly compactly, and in particular, compared to our previous scheme^88^, there is only a single probe with an MMF, which transmits both the probe light and the collected scattered light. Recently, the MMF as micro-endoscopes has attracted much interest in study of embedded imaging^89–91^. Similarly, the AF-RDV could be further developed along this way to enable in vivo sensing using the human-hair-width fiber probe. For this case, the collimator could be replaced with a miniature one, the potential candidates of which could be a microsphere lens or gradient-index (GRIN) lens glued to the fiber end. On the other hand, the AF-RDV also has distinct advantages in engineering and industrial scenarios. For example, it is promising to be a handheld equipment for motion monitoring, as well as allow remote measurements by extending the length of MMF. For the conventional laser sensing techniques with non-contact measurements of rotational speed^92–94^, generally, the local linear velocity is a parameter of direct acquisition and then the angular velocity is derived. As a comparison, the AF-RDV, with a direct method of angular velocity measuring, is more applicable for various targets including both macroscopic and microscopic objects.

The available range of the AF-RDV for measuring rotational speed is mainly dependent upon the response bandwidth of the PD and the mode order sculpted by the MSC. For example, the PD employed in the experiment is an amplified detector (Thorlabs, PDA50B2) whose bandwidth is switched to 22 kHz for suitable gain. Correspondingly, the maximum detectable rotational speed is 1.1 × 10^4^ rps. The upper limit of the measurement can be improved by employing an avalanche photodiode (APD) with typical MHz-level bandwidth. This means that the AF-RDV is expected to be used to monitor high-speed rotation in the study of wind tunnels, detonation waves, and particle physics. As for the weak motion, the AF-RDV needs to reach higher frequency resolution at the cost of measuring duration. This is because the frequency resolution is the inverse of sampling time, and the detected Doppler shifts are only twice compared to the rotational frequency due to the 1-order MSC employed. The fast response and measurement precision could be further improved by using higher-order fiber-based vortex generators. Another technical improvement of the AF-RDV that needs to be considered is the quick adjustment for coaxially aligned incidence. The mechanical manual translation/rotation stages are used to align the center of incident light and the center of spinning rough surface in the experiment, but in contrast, the requirement in practical applications is often non-manual automatic real-time alignment. A solution to this could be to realize scanning of the illuminated position with gratings by tuning the laser wavelength based on the achromatic property of the AF-RDV.

In summary, we have proposed and developed an AF-RDV with fiber-based elements for detecting angular velocity of the scatters based on the RDE. We have demonstrated the reciprocal character of the RDE by the identical Doppler shift extracted from the exchange of illuminating mode and detected mode. This further supports the fact that the RDE is related to the mode change during scattering rather than the initial mode of the probe light. The MSC, as a crucial element in the demonstration, takes the advantage of multi-functional operations including mode sculpting and filtering, which are implemented separately by other light-controlling devices like the SLM and vortex plates. We have also shown the achromatic property of the RDE by scanning the incident wavelength, enabling the AF-RDV within an ultra-broadband operating range. The observed harmonic peaks under broadband illumination suggest that the rotational Doppler shifts carried by different modes cannot be superimposed on each other, i.e., the mode-changing dependent property of the RDE. From the physical perspective, the achromatic and mode-changing dependent properties are the intrinsic nature that distinguishes the RDE from its linear counterpart. This facilitates the separation of rotational Doppler shifts from the mixtures with linear Doppler shifts for detecting a more complex motion (see ref. ^95^ for demonstration). With the goal of practicality, we would also construct a prototype of AF-RDV using a packaged MSC and other commercial components. It would be competitive for its cost-effective and compact advantages. For the application aspects, the AF-RDV may provide an exciting new practical sensing instrument for monitoring angular motion, with significant prospects in engineering, physics, and life sciences.

## Materials and methods

### Principle and fabrication of mode-selective coupler (MSC)

**Fig. 5:**
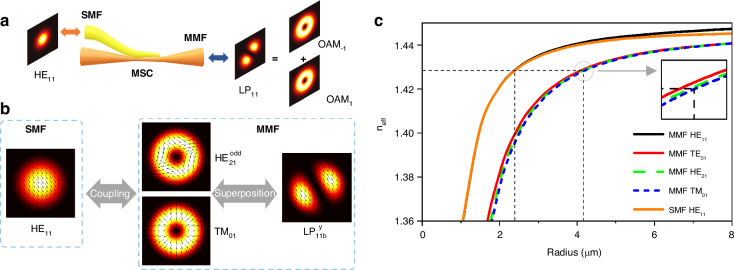
Principle of MSC. **a** The MSC consists of a standard SMF and a conventional MMF that are tapered together to enable mode-sculpting between Gaussian mode and superposed vortices. **b** The evolution of modes in MSC. **c** The effective refractive index (*n*_eff_) of different modes in SMF (HE_11_) and MMF (HE_11_, TE_01_, HE_21_, and TM_01_) as a function of the tapered radius of fiber

The MSC is designed and fabricated using a standard SMF (SMF-28) and a conventional MMF (OM3) to enable mode-sculpting between Gaussian mode and superposed vortices (OAM_±1_), as shown in Fig. 5a. For the superposed vortices with linear polarization, they appear as linearly polarized (LP) modes in weekly guiding fibers, i.e., ${\rm{LP}}_{11}={\rm{OAM}}_{+1}+{\rm{OAM}}_{-1}$. The mode-sculpting process of the MSC is shown in Fig. 5b. When the phase-matching condition is satisfied, the fundamental mode ${\rm{HE}}_{11}$ in the SMF would excite two degenerate vector modes ${\rm{HE}}_{21}^{{\rm{odd}}}$ and ${\rm{TM}}_{01}$ in the MMF. Particularly, different mode bases can be converted to each other in waveguides, such as the relation between vector modes and LP modes, ${\rm{TM}}_{01}+{\rm{HE}}_{21}^{{\rm{odd}}}={\rm{LP}}_{11{\rm{b}}}^{\rm{y}}$. As a result, a y-polarized ${\rm{LP}}_{11}$ mode is synthesized by the superposition of two vector modes in MMF. According to the mode-coupling theory, the phase-matching condition here means that the fundamental mode in SMF matches the effective refractive index (*n*_eff_) of the corresponding higher-order mode in MMF. The *n*_eff_ of different modes in SMF (HE_11_) and MMF (HE_11_, TE_01_, HE_21_ and TM_01_) as a function of the radius of fiber is calculated, as shown in Fig. 5c. This suggests that the *n*_eff_ of each mode decreases with the decreasing fiber radius in the tapering process. To reach the *n*_eff_ -matching, the radius of SMF needs to be reduced first by the pre-tapering process, followed by twisting it with MMF together and then performing fused-tapering process. Through the coincidence of *n*_eff_ curves, the HE_11_ mode in SMF is effectively coupled into the MMF as degenerate modes HE_21_ and TM_01_, while the TE_01_ mode is less excited due to a *n*_eff_ difference greater than 10^−4^, as shown in zoom-in details of Fig. 5c. Consequently, for the MSC, the superposed vortices would output from MMF when a Gaussian mode is launched into SMF, and conversely, it is also feasible for the reversed input and output. The insertion loss of the fabricated MSC for mode-sculpting is around 3 dB.

## Data Availability

The data underlying the results presented in this paper are available from the corresponding author upon reasonable request.
